# To Find a Better Dosimetric Parameter in the Predicting of Radiation-Induced Lung Toxicity Individually: Ventilation, Perfusion or CT based

**DOI:** 10.1038/srep44646

**Published:** 2017-03-15

**Authors:** Lin-Lin Xiao, Guoren Yang, Jinhu Chen, Xiaohui Wang, Qingwei Wu, Zongwei Huo, Qingxi Yu, Jinming Yu, Shuanghu Yuan

**Affiliations:** 1 School of Medicine and Life Sciences, University of Jinan-Shandong Academy of Medical Sciences, Jinan, Shandong, China; 2 Shandong Cancer Hospital and Institute- Shandong Cancer Hospital affiliated to Shandong University, Jinan, Shandong, China; 3 Shandong Academy of Medical Sciences, Jinan, Shandong, China

## Abstract

This study aimed to find a better dosimetric parameter in predicting of radiation-induced lung toxicity (RILT) in patients with non-small cell lung cancer (NSCLC) individually: ventilation(V), perfusion (Q) or computerized tomography (CT) based. V/Q single-photon emission computerized tomography (SPECT) was performed within 1 week prior to radiotherapy (RT). All V/Q imaging data was integrated into RT planning system, generating functional parameters based on V/Q SPECT. Fifty-seven NSCLC patients were enrolled in this prospective study. Fifteen (26.3%) patients underwent grade ≥2 RILT, the remaining forty-two (73.7%) patients didn’t. Q-MLD, Q-V20, V-MLD, V-V20 of functional parameters correlated more significantly with the occurrence of RILT compared to V20, MLD of anatomical parameters (r = 0.630; r = 0.644; r = 0.617; r = 0.651 vs. r = 0.424; r = 0.520 p < 0.05, respectively). In patients with chronic obstructive pulmonary diseases (COPD), V functional parameters reflected significant advantage in predicting RILT; while in patients without COPD, Q functional parameters reflected significant advantage. Analogous results were existed in fractimal analysis of global pulmonary function test (PFT). In patients with central-type NSCLC, V parameters were better than Q parameters; while in patients with peripheral-type NSCLC, the results were inverse. Therefore, this study demonstrated that choosing a suitable dosimetric parameter individually can help us predict RILT accurately.

Radiotherapy (RT) is an important treatment modalities for patients with non–small cell lung cancer (NSCLC)^1^ and the treatment success is often limited by the occurrence of radiation-induced lung toxicity (RILT) which is a common dose-limiting complication^2^. How to predict the high risk of RILT accurately is still difficult in present clinical practice. Anatomical mean lung dose (MLD) and V20 (relative volume of lung receiving more than 20 Gy dose) based on computerized tomography (CT) were considered as currently well-established means for predicting RILT and commonly used in the clinical medicine. With the in-depth study of pulmonary function, these conventional predictive dosimetric parameters have exposed more and more disadvantages.

Ventilation (V)/perfusion (Q) single-photon emission computerized tomography (SPECT) is an imaging modality which can be applied in the optimization of RT plans to identify ventilated and perfused regions of functional lung (FL) contributing to gas exchange and blood flow^3^. Some studies have related the incidence of RILT to the functional dosimetric parameters based on Q-SPECT^4^,^5^,^6^,^7^,^8^,^9^,^10^ which adds more accurate predictive value than that on anatomical CT. V-SPECT predicting RILT were rarely reported in previous studies, while a few researches have indicated that the use of V-SPECT can be used for guiding radiation beam arrangement in NSCLC^11^,^12^,^13^. Our prior study^14^ demonstrated that the V defects and Q defects were mismatched in some patients with NSCLC, and V/Q-SPECT would thus provide a more comprehensive pulmonary function assessment for their application in RT planning.

The purpose of this study was to find better a dosimetric parameter in the predicting of RILT in patients with NSCLC individually: V-SPECT, Q-SPECT or CT based.

## Results

### Patients Characteristics and Follow-up RILT

57 NSCLC patients were enrolled in this prospective study. All patients gave written consent to participate in this study, which was approved by the ethics committee of Shandong cancer hospital affiliated to Shandong University. Patients characteristics were given in Table 1. Fifteen (26.3%) patients underwent grade ≥2 RILT, the remaining forty-two (73.7%) patients didn’t. There are no significantly difference between the RILT patients and the non-RILT patients in patients characteristics.

### The Statistical Analysis Results

**Figure 1:**
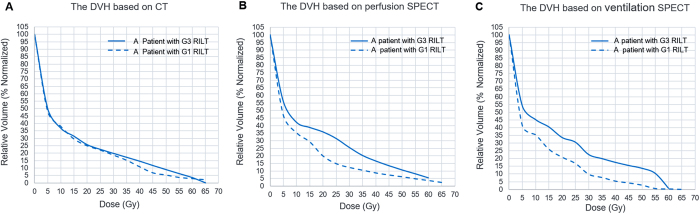
(**A**) The comparison of anatomical DVH based on CT in a patient with G3 (Grade 3) RILT and a patient with G1(Grade 1) RILT. (**B**) The comparison of functional DVH based on perfusion SPECT in a patient with G3 RILT and a patient with G1 RILT. (**C**) The comparison of functional DVH based on ventilation SPECT in a patient with G3 RILT and a patient with G1 RILT. DVH, dose volume histogram; CT, computerized tomography; RILT, radiation-induced lung toxicity; SPECT, single-photon emission computerized tomography.

**Figure 2:**
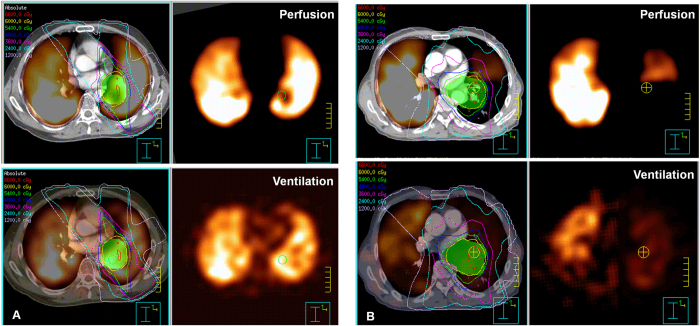
(**A**) The RT plannings and V/Q functional regions distribution of a patient with G3 (Grade 3) RILT. (**B**) The RT plannings and V/Q functional regions distribution of a patient with G1 (Grade 1) RILT. RILT, radiation-induced lung toxicity.

The Q-MLD, Q-V20, V-MLD, V-V20 of functional parameters correlated more significantly (Table 2) with the occurrence of RILT compared to V20, MLD of anatomical parameters (r = 0.631; r = 0.644; r = 0.617; r = 0.651 vs. r = 0.424; r = 0.520 p < 0.05, respectively). An comparison of anatomical and functional dose volume histogram (DVH) between one patient with G3 (Grade 3) RILT and one patient with G1 (Grade 1) RILT was shown on Fig. 1. In the anatomical DVH, both patients’ V20 was 25%; while in the functional DVH, Q-V20 was 35% for the patient with RILT, and 20% for the patient without RILT, V-V20 was 33% for the patient with RILT, and 22% for the patient without RILT. The RT plannings and V/Q functional regions distribution of the two patients were shown on Fig. 2. The V/Q functional regions of the patient with G3 (Grade 3) RILT has went much more radiation exposure than that of the patient with G1 (Grade 1) RILT.

**Figure 3:**
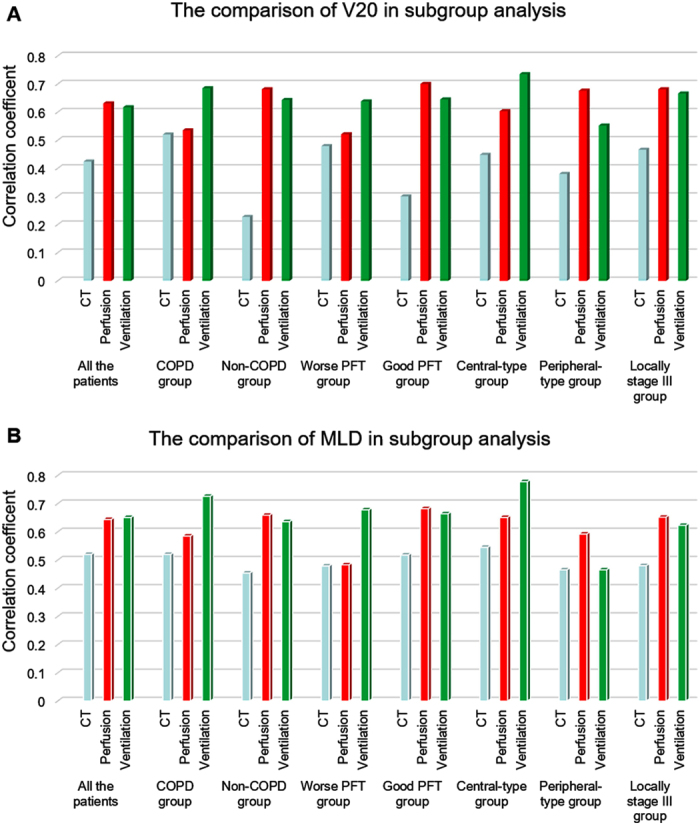
The comparison of correlation coefficient between RILT and dosimetric parameters (**A**, V20; **B**, MLD) among CT, perfusion SPECT and ventilation SPECT in subgroup analysis. RILT, radiation-induced lung toxicity; V20, relative volume of lung receiving more than 20 Gy dose; MLD, mean lung dose; CT, computerized tomography; SPECT, single-photon emission computerized tomography.

The further correlation analysis (Table 2 and Fig. 3) was carried out. The results showed that in patients with chronic obstructive pulmonary diseases (COPD), the correlation of V parameters with RILT was stronger than that of Q parameters and anatomical parameters(V-V20 vs.Q-V20 vs.V20: 0.685 vs.0.535 vs.0.520; V-MLD vs.Q-MLD vs.MLD: 0.726 vs.0.585 vs.0.520); in patients without COPD, V/Q functional parameters reflected similar advantage to anatomical parameters(V-V20 vs.Q-V20 vs.V20: 0.643 vs.0.681 vs.0.227; V-MLD vs.Q-MLD vs.MLD: 0.636 vs.0.659 vs.0.454).

The analogous results were existed in fractimal analysis of global pulmonary function test (PFT), in patients with worse PFT, V functional parameters were better than Q functional parameters and anatomical parameters (V-V20 vs.Q-V20 vs.V20: 0.638 vs.0.521 vs.0.479; V-MLD vs.Q-MLD vs.MLD: 0.678 vs.0.483 vs.0.479); in patients with good PFT, V/Q functional parameters reflected the similar advantage to anatomical parameters(V-V20 vs.Q-V20 vs.V20: 0.645 vs.0.700 vs.0.300; V-MLD vs.Q-MLD vs.MLD: 0.664 vs.0.682 vs.0.518).

In patients with central-type NSCLC, the correlation of V parameters with RILT was stronger than that of Q parameters which were better than anatomical parameters (V-V20 vs.Q-V20 vs.V20: 0.735 vs.0.603 vs.0.448; V-MLD vs. Q-MLD vs.MLD: 0.778 vs.0.651 vs.0.545); in patients with peripheral-type NSCLC, the results were different, Q functional parameters were better than V parameters which were better than anatomical parameters (Q-V20 vs.V-V20 vs.V20: 0.676 vs.0.552 vs.0.380; Q-MLD vs.V-MLD vs. MLD: 0.592 vs.0.465 vs.0.465).

There is no statistical difference in patients with medically inoperable stage I to II disease; while in patients with locally advanced stage III disease, V/Q functional parameters reflected the similar advantage to anatomical parameters(V-V20 vs.Q-V20 vs.V20: 0.666 vs.0.681 vs.0.446; V-MLD vs.Q-MLD vs.MLD: 0.623 vs.0.652 vs.0.480). There is no statistical significance in the analysis of age and pathology.

## Discussion

In this study, we compared the predictive value of functional dosimetric parameters based on V/Q SPECT and anatomical dosimetric parameters based on CT for RILT in patients with NSCLC. The results indicated that the correlation of V/Q functional parameters with RILT were similar, both stronger than that of anatomical parameters. The further fractimal analysis indicated that in patients with chronic obstructive pulmonary diseases (COPD) and patients with worse global pulmonary function test (PFT), V functional parameters reflected the biggest advantage in predicting RILT; while in patients with good PFT or in patients without COPD, Q functional parameters reflected the biggest advantage. In addition, in patients with central-type NSCLC, the correlation of V parameters with RILT was stronger than that of Q parameters; while in patients with peripheral-type NSCLC, the result was inverse.

A few decades ago, some researches had studied the correlation between SPECT and thoracic RT^15^,^16^,^17^,^18^. The study of Marks *et al*.^15^ indicated that Q SPECT scans showed valuable effect in minimizing irradiation of FL, then easing pulmonary toxicity in patients, especially in patients with worse lung dysfunction. Presently, there are already some researches on the ability of SPECT parameters to predict RILT, especially Q-SPECT^4^,^5^,^6^,^7^,^8^,^9^,^10^. Farr *et al*.^7^ conducted a study on this field by using the method of functional segmentation and functional SPECT-weighted normalization, which demonstrated that functional parameters based on Q-SPECT were better in predicting the risk of RILT compared to anatomical parameters based on CT. Since we reported that V/Q-SPECT provided a more comprehensive functional assessment and an additional value over Q-SPECT alone in assessing local pulmonary function^14^, the studies on both V/Q-SPECT images were more and more. Kimura *et al*.^6^ defined different functional lung region by using four-dimensional computed tomography (4D-CT) and V/Q-SPECT images and spearman rank correlation coefficient analysis showed that functional parameters correlated significantly with RILT, especially grade ≥3 RILT. Conventionally, we hypothesized Q-SPECT were better than V-SPECT in assessing pulmonary function and predicting RILT, while the specific and actual study was not found in the previous reports. So in this study, we compared the predictive value of dosimetric parameters based on V-SPECT, Q-SPECT and CT, then made the further fractimal analysis according to the different characteristics of the patients enrolled.

In patients with COPD, V parameters had stronger correlation with RILT comparing to Q parameters. This maybe due to the bad ventilatory function in patients with COPD. As the prior study^14^ demonstrated, in patients with COPD, especially with advanced (stage III–IV) COPD, there was V/Q discrepancy in some regions with almost complete absence of V but visible Q activity. So we supposed that only the regions with matched visible V/Q-SPECT images were the really accurate functional lung tissue, which may have the better predictive ability for RILT. And it’s the same thing in the matter of V parameters having advantages in the central-type lung cancer. Central-type tumors may cause the endobronchial obstruction of main stem bronchus or lobe bronchus, which results in the worse ventilatory function. All of the above indicated again that in the aspect of assessing the pulmonary function, V/Q-SPECT is more comprehensive than Q-SPECT. While in the clinical practice, a patient perhaps cannot afford to pay both the two checks. Understanding the specifically suitable crowd of V/Q-SPECT can help us choose a better and properer method.

However, this study has several limitations as followed: (1) some little deviation may be existed in the fusion process of V/Q SPECT images into RT planning; (2) there are various definition methods of FL, although the method we used in this study is frequently-applied, we do not know whether the method is the best; (3) the prior study^19^ has demonstrated that pulmonary functions may change during the course of RT and the functional lung regions would change simultaneously, the simple prediction for RILT by baseline-V/Q SPECT images is not comprehensive.

This study compared the predictive value of dosimetric parameters based on V-SPECT, Q-SPECT and CT for RILT in patients with NSCLC. However, the additional value of clinical RT plans guided by V/Q-SPECT image over that by CT alone in preventing the risk of RILT was still not approved clinically. Further study of formulating functional RT plan according to the functional region on V/Q-SPECT and applying the functional RT plan to suitable patients is ongoing in our institution. In addition, several other techniques, such as magnetic resonance imaging (MRI), 4D-CT^20^,^21^,^22^ and [18F] Fluorodeoxyglucose (18F-FDG) positron emission tomography (PET)^23^ which are currently not available for widespread clinical use, may be associated with the risk of RILT. Meanwhile, the correlation between regional SPECT density changes during the course of RT and RILT needs to be affirmed. All above require further study and exploration^7^.

We hope and believe that prospective RT guided by image techniques can yield less pulmonary toxicity with better tumor control.

## Conclusions

This study compared the predictive value of functional dosimetric parameters based on V/Q SPECT and anatomical dosimetric parameters based on CT for RILT in NSCLC, which demonstrated that choosing a suitable dosimetric parameter individually can help us predict RILT more accurately. Further study of formulating functional RT plan according to the functional region on V/Q-SPECT and applying the functional RT plan to suitable patients is ongoing in our institution.

## Methods and Material

### Study Population

The patients enrolled performed V/Q SPECT within 1 week prior to RT. The patients with following characteristics were excluded from the study: small-cell lung cancer (SCLC), mixed small cell/non-small cell cancer, pericardial effusion, pregnant woman.

Our investigation of 57 patients was approved by the Shandong cancer hospital affiliated to Shandong University Ethical Committee and has, therefore, been performed in accordance with the ethical standards laid down in the 1964 Declaration of Helsinki. All persons gave their informed consent prior to their inclusion in the study.

### V/Q-SPECT Imaging and RT Planning

V/Q SPECT images were acquired as previously described^14^. The SPECT images were used only to provide qualitative assessment of the distribution of pulmonary function and they were not incorporated quantitatively into treatment planning. The RT plans really performed were generated only from the simulation CT 3 days before treatment. In all plans, total radiation dose was set 60 Gy- 66 Gy with 2 Gy per fraction, 26 patients were treated with concurrent chemotherapy, including 11 patients with Docetaxel + platinum, 6 patients with Pemetrexeddisodium + platinum, 9 patients with Gemcitabine + platinum, the other 31 patients were treated without concurrent chemotherapy. Clinically acceptable plans that fulfilled the criteria for corresponding restriction of doses to lung, heart, esophagus, spinal-cord, and normal tissue were achieved for patients enrolled.

### Functional Lung Definition and Dosimetric Evaluations

SPECT/CT images were transferred to the treatment planning system(TPS, Varian Medical Systems, Palo Alto, CA, USA) via DICOM. Perfusion functional lung was weighted by the perfusion SPECT beyond a threshold of 30% of the maximum radioactivity; the other parts of the lung were called non-functional lung. The method of functional region distribution based on V-SPECT images is the same as Q-SPECT. We integrated the V/Q-SPECT images respectively into RT plans, generating functional MLD and V20 based on V/Q functional region distribution as above: Q-MLD, Q-V20, V-MLD, V-V20.

### Follow-up and RILT Evaluation

Scheduled follow-up examinations were performed at 1, 3, 6, 9, 12 months after RT and pulmonary symptoms were observed prospectively. The RILT was diagnosed and graded according to the National Cancer Institute Common Toxicity Criteria, version 4.0^24^. Pulmonary complications related to other aetiologies were not considered as RILT. Patients enrolled were divided on the basis of pulmonary symptoms and CT images after completion of RT into RILT group (Grade ≥ 2) and non-RILT group (Grade ≤ 1).

### Statistical Analysis

Statistical analysis were performed using the SPSS 17.0 statistical package. Spearman rank correlation coefficient was used to evaluate correlations. A two-way significance level of 5% was considered to be statistically significant.

## Additional Information

**How to cite this article**: Xiao, L.-L. *et al*. To Find a Better Dosimetric Parameter in the Predicting of Radiation-Induced Lung Toxicity Individually: Ventilation, Perfusion or CT based. *Sci. Rep.* **7**, 44646; doi: 10.1038/srep44646 (2017).

**Publisher's note:** Springer Nature remains neutral with regard to jurisdictional claims in published maps and institutional affiliations.

## Figures and Tables

**Table 1:** Patient characteristics.

Characteristic	RILT	Non-RILT	P value
**Sex**			0.825
** Male**	11	32	
** Female**	4	10	
**Age**			0.143
** <70**	8	31	
** >70**	7	11	
**Histopathology**			0.755
** Squamous cell carcinoma**	11	29	
** Adenocarcinoma**	4	13	
**Clinical stage**			0.334
** I**	2	4	
** II**	4	5	
** III**	9	33	
**Primary tumor location**			0.799
** Central type**	8	24	
** Peripheral type**	7	18	
**COPD**			0.274
** Yes**	7	13	
** No**	8	29	
**PFTs**			0.472
** Worse**	5	10	
** Good**	10	32	
**Concurrent chemotherapy**			0.517
** No**	7	24	
** Docetaxel + platinum**	2	9	
** Pemetrexeddisodium + platinum**	2	4	
** Gemcitabine + platinum**	4	5	

**Table 2:** Correlation of anatomical and functional parameters with incidence of RILT.

Parameters	r (P value)	Parameters	r (P value)	Comparison
All the patients				Q ≈ V > CT
V20	0.424(.063)	MLD	0.520(.042)	
Q-V20	0.631(.056)	Q-MLD	0.644(.026)	
V-V20	0.617(.014)	V-MLD	0.651(.017)	
COPD group				V > Q ≈ CT
V20	0.520(.049)	MLD	0.520(.049)	
Q-V20	0.535(.043)	Q-MLD	0.585(.047)	
V-V20	0.685(.039)	V-MLD	0.726(.033)	
Non-COPD group				V ≈ Q > CT
V20	0.227(.652)	MLD	0.454(.055)	
Q-V20	0.681(.001)	Q-MLD	0.659(.031)	
V-V20	0.643(.038)	V-MLD	0.636(.003)	
Worse PFT group				V > Q ≈ CT
V20	0.479(.041)	MLD	0.479(.053)	
Q-V20	0.531(.028)	Q-MLD	0.483(.001)	
V-V20	0.628(.022)	V-MLD	0.678(.036)	
Good PFT group				V ≈ Q > CT
V20	0.300(.304)	MLD	0.518(.033)	
Q-V20	0.700(.002)	Q-MLD	0.682(.040)	
V-V20	0.645(.014)	V-MLD	0.664(.015)	
Central-type group				V > Q > CT
V20	0.448(.027)	MLD	0.545(.004)	
Q-V20	0.603(.001)	Q-MLD	0.651(.046)	
V-V20	0.735(.038)	V-MLD	0.778(.022)	
Peripheral-type group				Q > V ≈ CT
V20	0.380(.078)	MLD	0.465(.042)	
Q-V20	0.676(.033)	Q-MLD	0.592(.016)	
V-V20	0.552(.039)	V-MLD	0.465(.002)	
Locally stage I and II group				V ≈ Q ≈ CT
V20	0.174(.631)	MLD	0.435(.209)	
Q-V20	0.348(.324)	Q-MLD	0.435(.209)	
V-V20	0.435(.209)	V-MLD	0.609(.062)	
Locally stage III group				V ≈ Q > CT
V20	0.466(.035)	MLD	0.480(.041)	
Q-V20	0.681(.026)	Q-MLD	0.652(.004)	
V-V20	0.666(.023)	V-MLD	0.623(.018)	
